# Genome Sequences of Community SARS-CoV-2 B.1.526 and P.1 Variants Circulating in the Dominican Republic

**DOI:** 10.1128/MRA.00744-21

**Published:** 2021-10-07

**Authors:** Robert Paulino-Ramirez, Victor Virgilio Calderon, Alejandro Vallejo Degaudenzi

**Affiliations:** a Instituto de Medicina Tropical and Salud Global, Universidad Iberoamericana (UNIBE), Santo Domingo, Dominican Republic; DOE Joint Genome Institute

## Abstract

Nearly complete genome sequences were obtained for a severe acute respiratory syndrome coronavirus 2 (SARS-CoV-2) variant of concern and two variants of interest from nasopharyngeal swab samples obtained during surveillance activities in urban communities, among individuals with no previous travel history, in Santo Domingo, Dominican Republic.

## ANNOUNCEMENT

The first severe acute respiratory syndrome coronavirus 2 (SARS-CoV-2) case in the Dominican Republic (DR) was reported on 29 February 2020 (1, 2). SARS-CoV-2 variants have been causing global health concerns due to their higher transmissibility, vaccine neutralization, and rapid accumulation of spike (S) protein mutations over time (3). Early in the pandemic, a D614G mutation was associated with high viral loads and increased transmissibility and was responsible for the first identified community transmission in the DR (4). This virus belongs to the *Betacoronavirus* genus of the family *Coronaviridae*, the members of which have been emerging as global threats since SARS-CoV was first recognized in 2002 (5).

**FIG 1:**
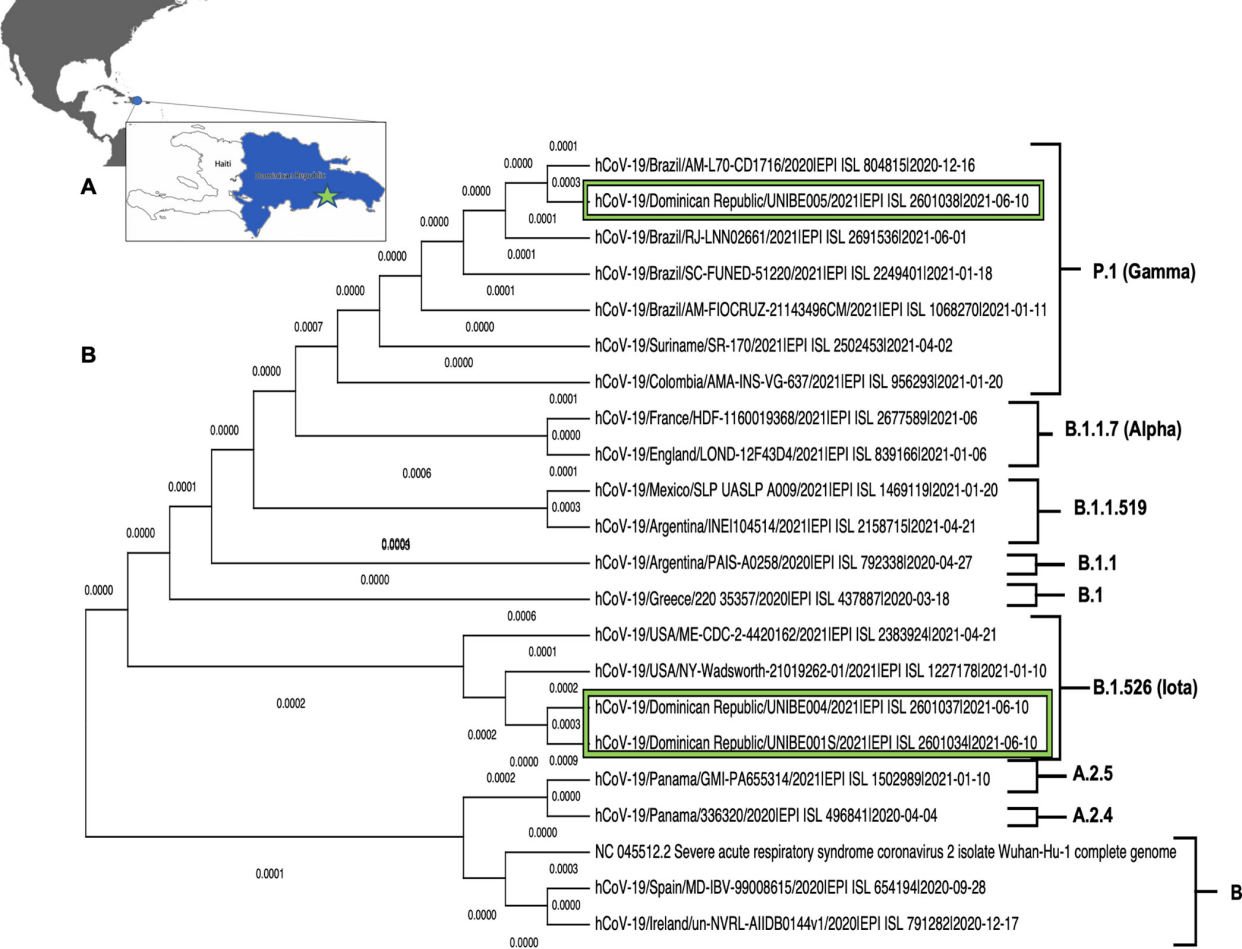
(A) Sample collection site (green star) in Santo Domingo, DR. Maps were created using ArcGIS and modified using the Sketches app to depict provinces where samples were obtained. (B) Phylogenetic tree of two variants of interest (B.1.526 [Iota]) and one variant of concern (P.1 [Gamma]) detected in DR (green rectangle) with relevant genomes (GISAID accession numbers are indicated). This tree was built with the maximum likelihood algorithm (GTR+G model) using MEGA X.

Here, we report nearly complete genome sequences for three samples of two SARS-CoV-2 variants circulating in the DR. Nasopharyngeal swab samples were obtained from symptomatic individuals suspected to be infected with SARS-CoV-2 during community surveillance interventions in the city of Santo Domingo, DR (Fig. 1A). This study was approved by the Universidad Iberoamericana (UNIBE) institutional review board (approval number CEI-2020-16) and the National Bioethical Committee (CONABIOS) (approval number 020-2021).

Samples were processed at the Instituto de Medicina Tropical y Salud Global (IMTSAG)-UNIBE for SARS-CoV-2 RNA amplification. RNA was extracted and purified with a 96-well automated nucleic acid extractor (Biocomma, Shenzhen, China) using the Biocomma viral DNA/RNA purification kit, following the manufacturer’s instructions. For the extraction procedure, 300 μl of sample was utilized, and subsequent amplification of these samples was carried out on an ABI 7500 Fast system (Applied Biosystems, USA). For the real-time quantitative PCR (RT-qPCR) protocol, we utilized the SARS-CoV-2 detection kit (BGI, Beijing, China), following the manufacturer’s instructions for both reagent volumes and the RT-qPCR thermocycling configuration. Samples were later selected based on cycle threshold (*C_T_*) values (*C_T_* values of <30) to reduce high-concentration inhibition during sequencing. Libraries were prepared according to the third iteration of the Artic Network protocol on SARS-CoV-2 for Oxford Nanopore Sequencing (https://artic.network/ncov-2019). The Artic Network protocol was reproduced completely except for the sequencing run time, for which we chose a value of 24 h so that all regions were represented in our data set. Data processing began with base calling of the raw fast5 data produced by the sequencer. Base calling was performed with high accuracy with Guppy base caller v5.0.7 after the sequencing. The run configuration was -c dna_r9.4.1_450bps_fast.Cfg. Demultiplexing and read filtering were performed so that the barcodes at each end had a read length between 400 and 700 bp. Demultiplexed and filtered reads were then processed through the Artic Network MinION pipeline (https://github.com/artic-network/fieldbioinformatics), ensuring further read filtering, primer trimming, amplicon coverage normalization, variant calling, and consensus building. Genomes obtained were visually curated with the IGV genome browser (6). Consensus genomes obtained from these processes were compared using the Nextstrain clade tool (7), with which the proper clade was identified. To perform phylogenetic analysis, other relevant genomes were downloaded from the GISAID database, the genomes were aligned with MAFFT v7 (https://mafft.cbrc.jp/alignment/server), and a phylogenetic tree was constructed (Fig. 1B) with a maximum likelihood algorithm based on the GTR model using MEGA X v7.0.26 (https://www.megasoftware.net). We used default parameters for all software.

Two genomes of these three viral samples correspond to the B.1.526 (Iota, 20C) lineage, which was first described in the New York region (8), and one to P.1 (Gamma, 20J/501Y.V3), which was detected first in Japan and later in Brazil, where it has become the dominant circulating variant (9). Genomic characteristics are presented in Table 1. The development of variants of SARS-CoV-2 is and will be quintessential as a result of natural selection; therefore, variant surveillance is of utmost importance to strengthen nonpharmaceutical interventions and to envision the negative impact on vaccines campaigns in low- and middle-income countries.

**TABLE 1:** Bioinformatic details for each sequence

Parameter	Data for variant with genome identification no.:		
	202106001S	202106004S	202106005S
SRA accession no.	SRR15697814	SRR15697813	SRR15697812
GISAID clade	GH/253G.V1	GR/501Y.V3	GH/253G.V1
PANGO lineage	B.1.526	P.1	B.1.526
WHO classification^*a*^	VOI (Iota)	VOC (Gamma)	VOI (Iota)
No. of raw reads	80,557	33,553	37,553
Genome size (bp)	29,882	29,894	29,898
No. of spots	41,725	34,007	88,091
Mutations in S protein^*b*^	4 mutations: A701V, D253G, D614G, E484K; 3 deletions: NSP6 F108del, NSP6 G107del, NSP6 S106del	2 mutations: T1027I, V1176F; 3 deletions: NSP6 F108del, NSP6 G107del, NSP6 S106del	7 mutations: A701V, D253G, D614G, E484K, H69Y, L5F, T95I; 3 deletions: NSP6 F108del, NSP6 G107del, NSP6 S106del
Read length (range [avg]) (bp)	480–550 (550)	460–550 (550)	460–550 (550)
Coverage (%)	96.49	80.61	95.31
GC content (%)	40	41.43	39.8

a WHO SARS-CoV-2 variant classification: VOI, variant of interest; VOC, variant of concern.

b NSP, nonstructural protein.

### Data availability.

Sequences of the SARS-CoV-2 variants were deposited in the NCBI database (GenBank accession numbers MZ645039, MZ576192, and MZ621913 and BioSample accession numbers SAMN21197472, SAMN21197473, and SAMN21197474) and the GISAID database (https://www.gisaid.org) (accession numbers EPI_ISL_2601034, EPI_ISL_2601037, and EPI_ISL_2601038). The raw reads were deposited in the NCBI Sequence Read Archive (SRA) database (SRA accession numbers SRR15697814, SRR15697813, and SRR15697812).
